# CPT1A mediated preservation of mitochondrial inhibits pyroptosis in pancreatic acinar cells

**DOI:** 10.3389/fcell.2025.1577669

**Published:** 2025-07-11

**Authors:** Yijiang Liu, Yangbo Liu, Xiuxian Yu, Simin Tian, Xiaojuan Li, Yu Gao, Xin Bao, Xiaoyi Wu, Boli Zhang, Wen Huang

**Affiliations:** ^1^ Department of Emergency Medicine, Natural and Biomimetic Medicine Research Center, Tissue-Orientated Property of Chinese Medicine Key Laboratory of Sichuan Province, West China School of Medicine, West China Hospital, Sichuan University, Chengdu, China; ^2^ Innovative Chinese Medicine Research Academician Workstation, West China Hospital, Sichuan University, Chengdu, China

**Keywords:** acute pancreatitis, CPT1A, mitochondrial dysfunction, pyroptosis, L-carnitine

## Abstract

**Introduction:**

Carnitine palmitoyltransferase 1A (CPT1A) is crucial for mitochondrial function, and its dysfunction has been linked to the development of several diseases. However, the role of CPT1A in severe acute pancreatitis (SAP) and its underlying mechanisms remain unclear. Mitochondrial damage-mediated pyroptosis has been identified as a critical factor in pancreatic acinar cell death during SAP. this study aimed to evaluate the protective role of CPT1A in SAP and investigate its association with pancreatic acinar cell pyroptosis.

**Methods:**

SAP models were established in male C57BL/6 mice by retrograde injection of 3% sodium taurocholate (STC) into the pancreatic duct and in primary acinar cells treated with 5 mM STC. Changes in Cpt1a mRNA and protein expression were assessed. Pancreatic pyroptosis was evaluated via activation of NLRP3 inflammasome-related proteins. Cpt1a was knocked down (siRNA) or inhibited (etomoxir) in cells. Cell viability was measured using Hoechst/PI staining, western blotting, and LDH release assays. The effects of CPT1A activators (C75, L-carnitine(LC)) on mitochondrial function (ΔΨm, mtROS, ox-mtDNA release) were examined in acinar cells.

**Results:**

In STC-induced SAP models (in vivo and in vitro), CPT1A expression was downregulated. Activating CPT1A with C75 or LC protected mitochondrial function (preserving ΔΨm, reducing mtROS, inhibiting ox-mtDNA release), thereby suppressing pyroptosis. LC treatment alleviated SAP in mice by inhibiting the NLRP3/GSDMD/Caspase-1 pathway and reducing acinar cell pyroptosis.

**Discussion:**

These findings reveal a novel protective mechanism of CPT1A in SAP. Enhancing CPT1A activity preserves mitochondrial functions and suppresses NLRP3/GSDMD-mediated pancreatic acinar cell pyroptosis, highlighting CPT1A as a potential therapeutic target.

## 1 Introduction

Acute pancreatitis (AP) is a severe inflammatory disorder of the pancreas, influenced by various factors, including gallstones, hyperlipidemia, and alcohol consumption (Habtezion et al., 2019; Mederos et al., 2021; Szatmary et al., 2022). Although most AP patients experience mild symptoms and spontaneous healing, approximately 20% may progress to severe acute pancreatitis (SAP), characterized by systemic inflammation and multiorgan dysfunction (Gukovskaya et al., 2017; Li et al., 2022; Szatmary et al., 2022). Despite significant research efforts, the intricate pathogenesis of SAP remains incompletely understood, and current therapeutic options are limited to supportive care, pain management, and nutritional support (Yokoe et al., 2015; Leppaniemi et al., 2019; Baron et al., 2020). Recent studies have shed light on the significant contribution of NLRP3 inflammasome-mediated pyroptosis in pancreatic acinar cell death during AP (Hegyi et al., 2011; Wang et al., 2020). Therefore, gaining a thorough understanding of the regulatory mechanism governing pancreatic acinar cell pyroptosis could lead to the identification of novel therapeutic targets for SAP treatment.

Pyroptosis is characterized by the activation of the NLRP3 inflammasome in damaged tissues. This triggers the release of pro-inflammatory cytokines and chemokines, which further exacerbate local tissue damage (He et al., 2016; Huang et al., 2021). Numerous cellular events, including intracellular ion dyshomeostasis (e.g., K^+^ and Ca^2+^), lysosomal damage, and mitochondrial dysfunction (e.g., mitochondrial reactive oxygen species (mtROS) and oxidized mitochondrial DNA (ox-mtDNA)), are involved in NLRP3 inflammasome activation (Kelley et al., 2019; De Gaetano et al., 2021; Huang et al., 2021). Recent studies have highlighted the significant role of NLRP3 inflammasome-mediated pyroptosis in the death of pancreatic acinar cells during AP (Gao et al., 2021; Sun et al., 2021; Wang J. H. et al., 2021; Wang X. Y. et al., 2021). However, the interrelationship and mechanisms underlying the interaction between mitochondrial function and the NLRP3 inflammasome in acinar cells have not been completely clarified.

Carnitine palmitoyltransferase 1A (CPT1A), a rate-limiting enzyme for fatty acid β-oxidation (FAO) and ATP production, is located in the outer mitochondrial membrane (Bonnefont et al., 2004; Schlaepfer and Joshi, 2020; Liang, 2023). Its deficiency or abnormal regulation has been linked to mitochondrial dysfunction, resulting in metabolic disorders and various types of cancer (Wang Y. N. et al., 2018; Schlaepfer and Joshi, 2020; Liang, 2023). Additionally, previous studies have shown that increased CPT1A expression can mitigate the loss of mitochondrial membrane potential in macrophage inflammation and significantly reduce the transcriptional expression of NLRP3 inflammasomes (Li et al., 2020). However, the role of CPT1A in the progression of SAP and its potential association with pyroptosis have not been explored.

L-carnitine (LC) is a naturally occurring compound that plays a vital role in transporting long-chain fatty acids to the mitochondrial matrix for β-oxidation, thereby providing energy (Adeva-Andany et al., 2017; Ferreira and McKenna, 2017; Virmani and Cirulli, 2022). Additionally, LC has been demonstrated to inhibit free radical generation, protecting mitochondria from lipid peroxidation under severe oxidative stress (Sima, 2007; Ferreira and McKenna, 2017; Wang Z. Y. et al., 2018). LC serves as a substrate of CPT1A and has been shown to enhance CPT1A expression and activity (Wang et al., 2021b; Chang et al., 2022; Li et al., 2023). Previous studies have shown that LC supplementation can alleviate AP progression in experimental models (McIlwrath et al., 2021), but the precise molecular mechanism remains unclear. We hypothesize that LC activates CPT1A, which may function as a key regulatory molecule mediating its protective effects by inhibiting pyroptosis in acinar cells.

In this study, we established a sodium taurocholate (STC)-treated SAP model and investigated the role of CPT1A in mitochondrial dysfunction and cell pyroptosis. Our results revealed that promoting CPT1A expression alleviated mtROS-mediated acinar cell pyroptosis through the NLRP3/GSDMD signaling pathway, ultimately improving SAP progression. These findings uncover the underlying mechanisms of CPT1A regulation in acinar cells and suggest it as a potential therapeutic target for SAP.

## 2 Materials and methods

### 2.1 Reagents and antibodies

L-carnitine (#C105423), purity >98%, was purchased from Leyan (Shanghai, China). Sodium taurocholate (STC, #551055) was purchased from J&K Scientific (Beijing, China). Etomoxir (#HY-50202), C75 (#HY-12364) and Triton WR-1339 (HY-B1068) were purchased from MedChemExpress (New Jersey, United States). Collagenase IV (#C9407) and DAPI (#D9542) were purchased from Sigma-Aldrich (St. Louis, MO, United States).

### 2.2 Animals

Male C57BL/6J mice were obtained from the animal feeding center of Sichuan University Health Science Center (Chengdu, China). All mice were housed in specific-pathogen-free (SPF) conditions with a controlled temperature (21°C ± 0.5°C), relative humidity (55% ± 1%), and on a 12-h light/12-h dark cycle. Before commencing the experiments, the mice were acclimatized for a minimum of 7 days and provided with *ad libitum* access to food and water. All experimental procedures were conducted in accordance with the ARRIVE guidelines and were approved by the Ethics Committee of West China Hospital at Sichuan University (Approval No. 20220218010).

### 2.3 Cell culture

Mouse pancreatic acinar cell line 266–6 (CRL-2151, ATCC, Manassas, VA, United States) was cultured in Dulbecco’s modified Eagle’s medium (DMEM; Gibco, America) supplemented with 10% fetal bovine serum and 1% penicillin-streptomycin at 37°C with 5% CO_2_. Cell line identity was validated by short tandem repeat profiling, and routine mycoplasma testing was negative for contamination.

### 2.4 Mouse SAP model establishment

The male C57BL/6J mice (weight 25–30 g) were randomly assigned to four groups (n = 5): the control (Control) group, the model (STC) group, the low LC plus STC (100 mg.kg^−1^ LC+STC) group, the high LC plus STC (200 mg.kg^−1^ LC+STC) group. Prior to SAP induction, LC was dissolved in saline and administered intragastrically at 100 mg.kg^−1^ per day (low LC group) or 100 mg.kg^−1^ LC per day (high LC group) for 1 week.

The mouse model of SAP was induced by retrograde injection of 3% STC into the pancreatic duct as previously described with some modifications (Perides et al., 2010). Briefly, the mice were anesthetized by intraperitoneal injection of 30 mg/kg pentobarbital sodium after fasting for 12 h. A midline laparotomy was performed to identify the pancreas and papilla of Vater. A 7-0 traction suture was used immobilized the duodenum. SAP was induced by occluding the proximal common bile duct at the liver hilum with a micro-clamp. The infusion pump delivered the 3% STC solution retrogradely into the bile-pancreatic duct via the papilla of Vater at the rate of 0.06 mL/h for 10 min. After the injection, all instruments were carefully removed, and the puncture site was meticulously sutured using an 8-0 prolene suture. The abdomen was closed in two layers using 6-0 prolene sutures. To prevent dehydration, 0.8 mL of normal saline was subcutaneously administered. Sham-operated mice underwent same surgical procedures without STC injection.

### 2.5 Biochemical analysis

Blood samples were collected and centrifuged at 3,000 rpm for 10 min to obtain the serum. The serum samples were stored at −80°C in case of untimely detection. The concentrations of serum amylase, lipase, total cholesterol and triglycerides were quantified utilizing a commercially available kit with an automatic biochemical analyzer (Roche, Germany), adhering closely to the provided manufacturer’s guidelines.

### 2.6 ELISA assay

The concentrations of IL-1β were detected using the enzyme-linked immunosorbent assay (ELISA) kit as the manufacturer’s instructions (Elabscience Biotechnology Co. Ltd., Wuhan, China) suggested. The optical density was detected by Synergy Mx multifunctional Microplate Reader (Gene Company Ltd., Hongkong, China).

### 2.7 Histopathological examination

The pancreas and lungs were promptly dissected following euthanasia and fixed in 4% paraformaldehyde for 24 h at room temperature. The fixed pancreatic and pulmonary specimens were embedded in paraffin, cut into 4 μm sections, and subjected to haematoxylin and eosin (H&E) staining. Two independent pathologists conducted a blinded assessment of pancreatic and lung injury histopathological indicators (including edema, inflammation, and necrosis), using a scale ranging from 0 to 3, as previously outlined (Osman et al., 1998; Wildi et al., 2007) (Supplementary Table S1). A minimum of seven randomly selected visual fields (×200) per slide were examined under an optical microscope (ZEISS, Germany). The severity of histopathological changes was quantified by calculating the average score for each tissue sample.

### 2.8 Molecular docking analysis

In silico investigations were undertaken to elucidate the binding mechanism between LC and CPT1A (Uniprot ID: P50416). The molecular structure of LC and the protein crystal structure of CPT1A were imported into Discovery Studio 2021 software (BIOVIA, Dassault Systèmes, San Diego, CA, United States), facilitating a comprehensive analysis. Subsequently, the docking procedure was executed employing the AutoDock optimization tool within the software platform.

### 2.9 Pancreatic primary acinar cells isolation

Freshly isolated pancreatic acinar cells from male C57BL/6J mice were obtained using a collagenase IV-based digestion procedure, as previously described (Shen et al., 2018). The mice were euthanized by cervical dislocation, and the pancreatic tissue was dissected. The tissue was then washed three times with PBS. Subsequently, collagenase IV solution (200 U/mL in HBSS buffer) was injected, and the tissue was incubated in a 37°C water bath for 19 min. The isolated cells were mechanically dispersed and filtered through a 100 μm cell strainer. The filtered cells were then centrifuged at 800 rpm for 2 min to obtain a cell pellet, which was subsequently dispersed in HBSS buffer solution. After isolation, the cells were treated at room temperature and tested within 4 h.

### 2.10 Cell transfection

We entrusted Youkang Biotechnology Co. Ltd. (Chengdu, China) to design and synthesize the small interfering RNAs (siRNA) to interfere with Cpt1a mRNA expression. Transfection (100 pmol/mL) was performed using LipofectamineTM RNAiMAX reagent. Overexpression of Cpt1a-Flag plasmid was obtained from Jingbai Biotechnology Co. Ltd. (Nanjing, China), and lipofectamine 3000 was used for the transfection. Cells were subsequently divided into different groups according to the experimental requirements. For siRNA transfection, the cells were divided into four experimental groups: NC (negative control) group (cells transfected with NC siRNA; NC + STC group (NC siRNA-transfected cells treated with STC); siCpt1a group (cells transfected with siCpt1a); siCpt1a + STC group (siCpt1a-transfected cells treated with STC). For overexpression plasmid transfection, the cells were divided into four groups: EV (empty vector) group (cells transfected with EV; EV + STC group (EV-transfected cells treated with STC); Cpt1a-OE group (cells transfected with Cpt1a overexpression plasmid); Cpt1a-OE + STC group (Cpt1a-OE-transfected cells treated with STC).

### 2.11 LDH release assay

Collect the supernatant or the culture medium of the cells. The LDH Cytotoxicity Assay Kit (YEASEN, China) was used to measure the LDH release. The procedure was performed according to the manufacturer’s instructions and measured at 490 nm using a microplate reader.

### 2.12 Hoechst/PI staining

The Hoechst/propidium iodide (PI) dual staining method was utilized to investigate the effects of LC, etomoxir, or C75 pretreatment on STC-induced primary acinar cell pyroptosis. Briefly, freshly isolated primary acinar cells were pretreated with the aforementioned reagents for 30 min, followed by 50 min of treatment with 5 mM STC. The treatments were conducted with gentle shaking at 60 rpm and a temperature of 37°C. For staining, Hoechst 33342 (50 μg/mL) and PI (1 μM) were employed to respectively label total nuclei and necrotic primary acinar cells with plasma membrane rupture. Image recording was performed using an automatic ZEISS AX10 imager A2/AX10 cam HRC (Jena GmbH, Germany). The total number of acinar cells exhibiting PI uptake was determined for each condition, with a minimum of 1,000 cells counted, providing the percentage of necrosis (%) for each group. The experimental setup involved five isolates per condition to ensure robust statistical analysis.

### 2.13 Measurement of ΔΨm

Measurement of mitochondrial membrane potential (ΔΨm) was conducted using JC-1 (#G009-1-1, Nanjing Jiancheng Bioengineering Institute, China) following the manufacturer’s instructions. JC-1 dye accumulates in active mitochondria, leading to the formation of J-aggregates with red/orange fluorescence emission at 590 nm, while under conditions of mitochondrial depolarization or low potentials, JC-1 exists as J-monomers emitting a green fluorescence at 525 nm. Primary acinar cells subjected to different treatments were subsequently incubated with JC-1 (7.5 μM) at 37°C for the final 20 min of the experiment. Following the incubation period, cells were washed twice with PBS and imaged using a fluorescence microscope (Nikon, Japan). The ratio of JC-1 aggregate to monomer (red/green) fluorescence density were analyzed using Image-Pro Plus 7 software (Media Cybernetics Inc., United States).

### 2.14 ATP assay

The release of ATP from primary acinar cells exposed to various treatments was assessed using the CellTiter-Lumi™ Luminescent Assay Kit, following the manufacturer’s instructions (C0065, Beyotime, China). Primary acinar cells in different treatment groups were centrifuged at 1,000 *g* for 5 min at room temperature. The supernatant containing released ATP was carefully transferred to a black 96-well plate and mixed with 100 μL of CellTiter-Lumi™ ATP detection reagent. The mixture was then incubated for 10 min at room temperature. To quantify the ATP release, luminescent signals were measured using a Synergy Mx multifunctional Microplate Reader (Gene Company Ltd., Hong Kong, China). The data were presented as relative ATP release by comparing it with the control group.

### 2.15 Mitochondrial ROS production

To evaluate mitochondrial ROS production, mitochondrial superoxide levels were quantified using the MitoSOX Red Mitochondrial Superoxide Indicator dye (Yeasen Biotechnology, 40778ES50). The primary acinar cells were subjected to the specified treatment conditions and subsequently incubated with mtSOX red at a concentration of 2 µM for a duration of 30 min at 37°C. Following the incubation period and subsequent washing steps, the fluorescence emissions were quantified using a Tecan Infinite 200 Pro Microplate reader. The obtained data are depicted as the mean intensity of MitoSOX fluorescence fold change compared to the control. For visualization of MitoSOX staining, the cells were examined utilizing a fluorescence microscope (Nikon, Japan).

### 2.16 Oxygen consumption rate assay

After treatment, OCR were measured using corresponding assay kits (Elabscience, Wuhan) according to the manufacturer’s instructions. The microplate was placed in a multifunctional microplate reader set at 37°C with dynamic reading mode. The excitation wavelength was set at 405 nm, the emission wavelength was set at 675 nm, and the detection was performed every 2 min for 90 min. Finally, the fluorescence values were plotted against time, and OCR was represented by the slopes of the curves.

### 2.17 Mitochondrial 8-hydroxydeoxyguanosine (8-OHdG) assay

The mitochondrial 8-OHdG contents, served as an indicator of mtDNA oxidative damage, were assessed using the 8-OHdG Check ELISA kit (Wuhan, China). Mitochondria were isolated from primary acinar cells exposed to different treatments using the Mitochondria Isolation Kit (Beyotime, China) following the manufacturer’s instructions. The isolated mitochondria were then resuspended in PBS and underwent three freeze-thaw cycles from 37°C to −80°C. Afterward, the mitochondrial suspensions were centrifuged at 2000×*g* for 20 min. Subsequently, 50 μL of the supernatant samples was collected for enzyme-linked immunosorbent assay. Data analysis was performed by measuring optical density (OD) values at a wavelength of 450 nm using a spectrumMax M5 microplate reader (Molecular Devices, LLC, Sunnyvale, CA, United States).

### 2.18 Immunofluorescence staining

Pacreatic paraffin sections (3 μm) were placed in a heat-incubator at 60°C for 2 h, and subsequently placed in xylene for 30 min at room temperature. The sections were then rehydrated in descending concentrations of ethanol and washed three times in deionized water. The sections were retrevalled and subsequently incubated with blocking buffer for 30 min (2% bovine serum albumin in PBS). Next, the sections were drop incubated with 50 μL primary antibody targeting NLRP3 (#BA3677, 1:50, BOSTER), GSDMD (AF4012, 1:100, Affinity), and CPT1A (#66039-1-Ig, 1:50, Proteintech) at 4°C overnight. The following day, the sections were washed three times in PBS and subsequently incubated with secondary antibodies and the nuclear stain DAPI for 1 h before being observed using a fluorescence microscope (Nikon, Japan).

### 2.19 RNA extraction and quantitative real-time PCR (qRT‒PCR)

The RNA was extracted from tissues and cells with Trizol reagent according to the manufacturer’s instructions. The RNA purity and concentration were analyzed using a Nanodrop 1000 spectrophotometer (Thermo Fisher, United States). cDNA was synthesized using a cDNA reverse transcription kit (YEASEN, China). The 2X qPCR SYBR Green Master Mix (YEASEN, China) was used for qRT‒PCR.

The primers used were as follows: Cpt1a-1, 5′-GGTCTTCTCGGGTCGAAAGC-3′ (forward) and 5′-TCCTCCCACCAGTCACTCAC-3′ (reverse); Cpt1a-2, 5′-CTCCGCCTGAGCCATGAAG-3′ (forward) and 5′-CACCAGTGATGATGCCATTCT-3′ (reverse); β-actin, 5′-GAGGTATCCTGACCCTGAAGTA-3′ (forward) and 5′-CACACGCAGCTCATTGTAGA-3′ (reverse).

### 2.20 Western blotting analysis

Protein lysates were prepared from pancreatic tissue or isolated primary acinar cells by homogenization in RIPA buffer containing protease and phosphatase inhibitors. 20 μg protein samples were separated on 10% or 12.5% SDS-polyacrylamide gels and transferred to PVDF membranes. Primary antibodies targeting specific proteins were employed at the following dilutions: CPT1A (1:1,000, Proteintech, #66039-1-Ig), NLRP3 (1:1,000, BOSTER, #BA3677), cleaved Caspase-1 (1:1,000, Cell Signaling Technology, #89332), GSDMD (1:1,000, Affinity, AF4012), β-Actin (1:10,000, ABclonal, #AC038), goat anti-rabbit (1:10,000, Proteintech, #SA00001-2) and goat anti-mouse (1:10,000, Proteintech, #SA00001-1). Protein bands were visualized using enhanced chemiluminescence (ECL) reagents (Millipore, United States) and imaged by a ChemiDoc MP imaging system (Bio-Rad, United States). β-Actin was utilized as a loading control. Data were collected and analyzed from at least three independent samples.

### 2.21 Statistical analysis

The data were presented as means ± SEM and were analyzed using GraphPad Prism (version 8.01, United States). Prior to conducting the Student’s t-test or one-way ANOVA, the data underwent testing for homogeneity of variance using the Shapiro-Wilk test or Levene test, respectively. A minimum of three independent experiments were conducted, and statistical significance was defined as a *p* value <0.05.

## 3 Results

### 3.1 Pancreatic CPT1A was downregulated in STC-induced SAP mice

**FIGURE 1:**
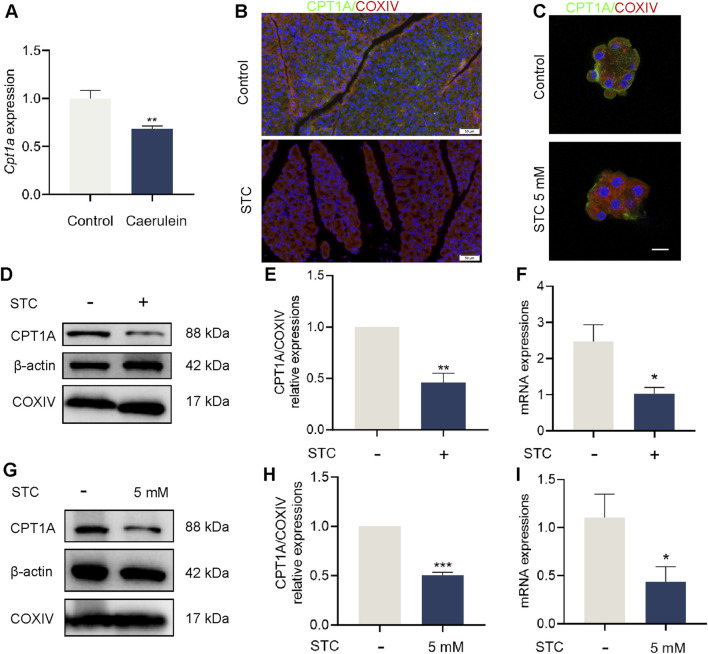
Pancreatic CPT1A was downregulated in STC-induced SAP mice. **(A)** Analysis of Cpt1a gene expression in dataset GSE3644. **(B)** Immunofluorescence staining of CPT1A in the pancreas of mice. Scale bar: 50 μm. **(C)** CPT1A fluorescence was observed in STC-treated primary acinar cells. Scale bar: 20 μm. **(D,E)** Protein levels of CPT1A, COXIV and β-actin in STC-treated pancreas tissue of mice. **(F)** Cpt1a mRNA expression in the pancreas of mice. **(G,H)** Protein levels of CPT1A, COXIV and β-actin in primary acinar cells in response to STC (5 mM). **(I)** Cpt1a mRNA expression in primary acinar cells. All data are presented as means ± SEM, n = 3–5. *p < 0.05, **p < 0.01, ***p < 0.001 vs. Control group. STC: sodium taurocholate.

Mitochondrial function in acinar cells plays a critical role in maintaining cellular energy balance and mitigating cellular damage during AP. CPT1A, a key enzyme involved in mitochondrial energy production, was investigated for its expression changes in SAP. To assess this, we first screened data from the GEO DataSets. Due to the absence of valid STC-induced AP datasets, we referred to the caerulein-induced AP model. Analysis of dataset GSE3644 revealed a downregulation of Cpt1a gene expression during AP (Figure 1A). Immunofluorescence staining of tissue paraffin sections showed a reduction in CPT1A-positive cells in pancreatic tissues of STC-induced SAP mice (Figure 1B; Supplementary Figure S1A). Additionally, decreased CPT1A fluorescence intensity was observed in STC-treated primary acinar cells (Figure 1C; Supplementary Figure S1B). To further confirm these findings, we performed Western blot and qRT-PCR analyses, which showed that both protein and mRNA levels of CPT1A were downregulated in STC-induced SAP models, both *in vitro* and *in vivo* (Figures 1D–I). These results strongly suggest that CPT1A is inhibited during SAP.

### 3.2 Inhibition of CPT1A exacerbates STC-induced cell death

**FIGURE 2:**
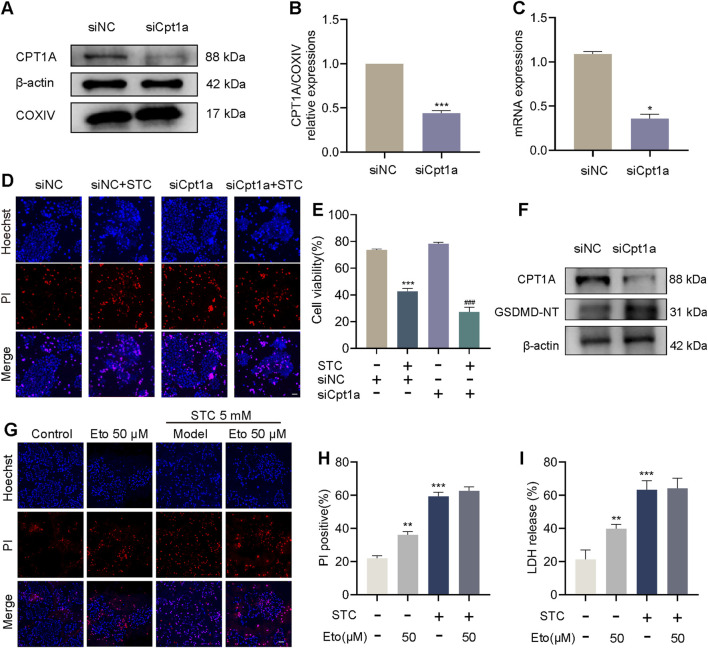
Inhibition of CPT1A exacerbates STC-induced cell death. Inhibition of CPT1A exacerbates STC-induced cell death. **(A,B)** Protein levels of CPT1A, COXIV and β-actin in 266-6 cells in response to siNC and siCpt1a. **(C)** Cpt1a mRNA relative expressions to control. **(D)** Representative images of Hoechst 33342 (blue) and PI (red) staining of 266-6 cells treated with STC, STC+siCpt1a, respectively. Scale bar: 50 μm. **(E)** CCK8 was used to determine the cell viability. **(F)** Protein levels of CPT1A and GSDMD-NT in 266-6 cells received different treatments. **(G)** Representative images of Hoechst 33342 (blue) and PI (red) staining of primary acinar cells received different treatments. Scale bar: 50 μm. **(H)** The ratio of PI positive cells in different group. **(I)** LDH release in the lysate of acinar cells. All data are presented as means ± SEM, n = 3–5. *p < 0.05, **p < 0.01, ***p < 0.001 vs. Control group or siNC group. ^###^ *p* < 0.001 vs. STC+siNC group. NC: negative control; STC: sodium taurocholate; Eto: Etomoxir.

To further investigate the role of CPT1A in STC-induced SAP, we transfected 266-6 cells with siRNA to reduce the basal expression of Cpt1a. The decreased expression of the CPT1A in 266-6 cells was confirmed by qPCR and Western blot analysis (Figures 2A–C). The Hoechst/PI dual staining and CCK-8 assays demonstrated that Cpt1a knockdown promoted cell death in 266-6 cells upon STC treatment (Figures 2D,E). We also transfected cells with a plasmid expressing Cpt1a to induce its overexpression (Supplementary Figure S2). The results revealed that Cpt1a overexpression significantly alleviated the reduction in cell viability induced by STC stimulation (Supplementary Figure S2D). NLRP3/GSDMD-mediated pyroptosis has been identified as a crucial contributor to acinar cell death during SAP (Wang J. H. et al., 2021; Al Mamun et al., 2022). Knockdown of Cpt1a increased GSDMD-NT expression in acinar cells (Figure 2F). Moreover, NLRP3 expression and IL-1β release were substantially reduced in Cpt1a-overexpressing cells treated with STC (Supplementary Figures S2E,G). Additionally, we assessed the effect of etomoxir, a selective CPT1A inhibitor, on STC-induced cell death in primary acinar cells using Hoechst/PI staining. As shown in Figures 2G,H, pretreatment with etomoxir (50 μM) significantly inhibited cell viability and LDH release (Figure 2I). Collectively, these findings suggest that CPT1A inhibition may exacerbates GSDMD-mediated pyroptosis.

### 3.3 CPT1A attenuates STC-induced cell pyroptosis through preserving mitochondrial function

**FIGURE 3:**
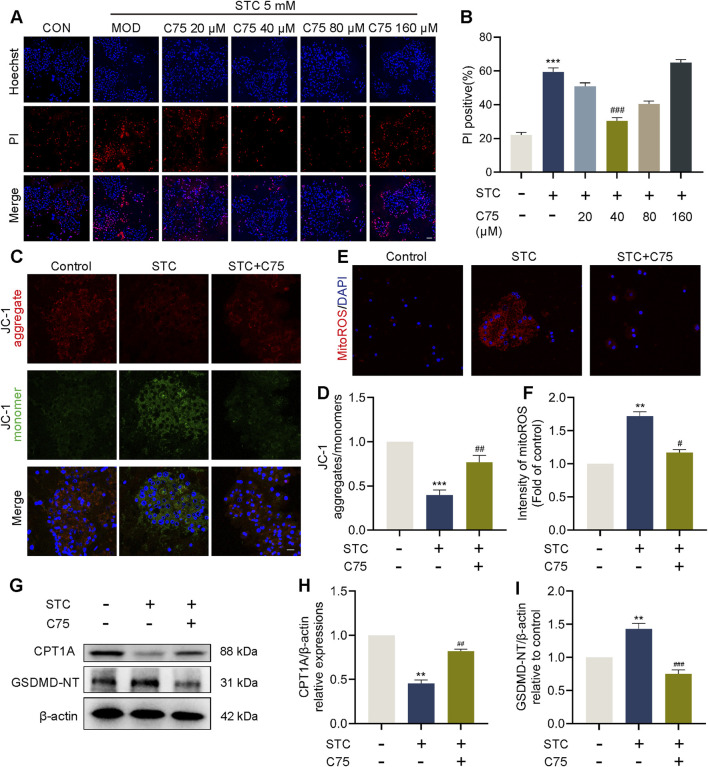
CPT1A attenuates STC-induced cell pyroptosis through preserving mitochondrial function. CPT1A attenuates STC-induced cell pyroptosis through preserving mitochondrial function. **(A,B)** Representative images and quantification of Hoechst 33342 (blue) and PI (red) staining of primary acinar cells received different concentrations of C75 treatments. **(C,D)** JC-1 fluorescent staining of primary acinar cells in different treatment groups. Scale bar: 50 μm. **(E,F)** Fluorescent quantification and imaging of mtROS in primary acinar cells in different treatment groups. **(G–I)** Protein levels of CPT1A and GSDMD-NT in the primary acinar cell received different treatments. β-actin served as the loading control (n = 3). All data are presented as means ± SEM, n = 3–5. *p < 0.05, **p < 0.01, ***p < 0.001 vs. Control group. ^#^ *p* < 0.05, ^##^ *p* < 0.01, ^###^ *p* < 0.001 vs. STC group.

Mitochondrial dysfunction, particularly the release of the mtROS, is an important trigger of NLRP3-dependent pyroptosis (Xian et al., 2022; Zhu et al., 2022). Given the critical role of CPT1A in maintaining mitochondrial function, we further explored whether the mechanism by which CPT1A inhibits NLRP3 inflammasome activation is linked to its regulatory effect on mitochondrial dysfunction. First, we pretreated primary acinar cells with C75 (a CPT1A agonist) and assessed its effects on STC-induced acinar cells using Hoechst/PI staining. As shown in Figures 3A,B, pretreatment with C75 (40 μM) exhibited a clear protective effect. We then evaluated mitochondrial function in acinar cells by examining mitochondrial membrane potential (ΔΨm, measured by JC-1) and mtROS production (Figures 3C–F). The results indicated that C75 pretreatment significantly mitigated the decrease in ΔΨm and the increase in mtROS in STC-treated primary acinar cells. Our results also showed that STC stimulation significantly decreased Oxygen Consumption Rate (OCR) in cells of the control group, suggesting impaired mitochondrial respiration. Activation of CPT1A by C75 partially restored the OCR levels in the cells (Supplementary Figure S3). Additionally, we used transmission electron microscopy (TEM) to further investigate the effects of C75 on mitochondria. According to previous studies (Del Dotto et al., 2017), mitochondria fall into different categories, including Class I (more than four cristae), Class II (two or three cristae), and Class III (no more than one cristae) and Class A (mitochondria with a dense matrix) and Class B (mitochondria with a hypodense matrix), we quantified these different categories. In the STC-treated group, mitochondrial swelling and reduced mitochondrial cristae were observed, along with smaller nuclei. In contrast, the C75 group exhibited reduced mitochondrial swelling and an increased number of mitochondrial cristae (Supplementary Figure S4). Fluorescent staining showed a decrease in nuclear size in the SAP cell model (Supplementary Figure S5). We further examined the expression of the related proteins SUN1/2 and observed a reduction in their levels (Supplementary Figure S6). Western blotting data further demonstrated that the expression of active forms of GSDMD (GSDMD-NT) was inhibited in STC-pretreated primary acinar cells with C75 (Figures 3G–I). Then we performed ELISA-based quantification of IL-1β using cell lysates. C75 treatment significantly inhibited the STC-induced increase in IL-1β levels, further supporting the suppression of NLRP3 inflammasome activation (Supplementary Figure S7).

### 3.4 LC protects against STC-induced acinar cell pyroptosis via CPT1A

**FIGURE 4:**
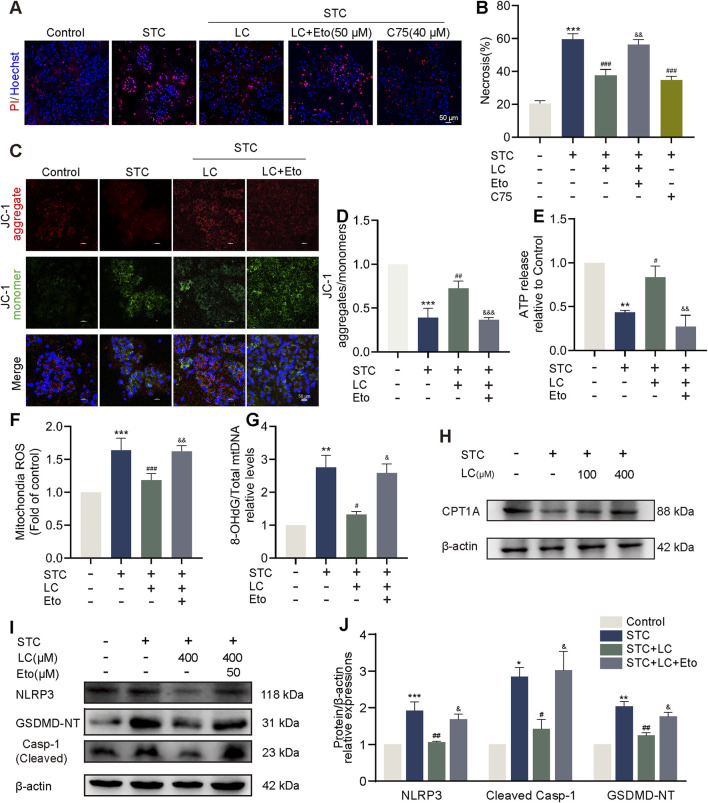
LC protects against STC-induced acinar cell pyroptosis via CPT1A. **(A,B)** Representative images and quantification of Hoechst 33342 (blue) and PI (red) staining of primary acinar cells received different treatments. Scale bar: 50 μm. **(C,D)** JC-1 fluorescent staining of primary acinar cells in different treatment groups. Scale bar: 50 μm. **(E)** The release of ATP levels in the supernatant of cultured primary acinar cells received different treatments. **(F)** Quantification of mtROS in primary acinar cells in different treatment groups. **(G)** The ratio of 8-OHdG to total mtDNA content in primary acinar cells received different treatments. **(H)** Protein levels of CPT1A and β-actin in the primary acinar cells received different treatments. **(I,J)** Protein levels of NLRP3, cleaved Caspase-1 and GSDMD-NT in the primary acinar cell received different treatments. β-actin served as the loading control (n = 3). Data are presented as means ± SEM, n = 3–5. *p < 0.05, **p < 0.01, ***p < 0.001 vs. Control group. ^#^ *p* < 0.05, ^##^ *p* < 0.01, ^###^ *p* < 0.001, vs. STC group. ^&^ *p* < 0.05, ^&&^ *p* < 0.01, ^&&&^ *p* < 0.001 vs. STC+LC treatment group. STC: sodium taurocholate; LC: L-carnitine; Eto: Etomoxir.

We observed that concentrations of C75 higher than 80 μM resulted in significant cytotoxicity (Figures 3A,B). Therefore, we turned to a safer CPT1A activator. Molecular docking analysis identified potential binding sites of LC on CPT1A (Supplementary Figure S8). Primary acinar cells were preincubated with various doses of LC (100–800 μM) before STC treatment. Hoechst/PI dual staining results showed that LC had no cytotoxicity, and its treatment significantly inhibited STC-induced acinar cell death in a dose-dependent manner within the 100–400 μM dose range (Supplementary Figure S9). Consequently, the most effective *in vitro* treatment dose of LC (400 μM) was selected for subsequent mechanism studies. As shown in Figures 4A,B, pretreatment with etomoxir (50 μM) effectively abolished the beneficial effect of LC on acinar cell death, while pretreatment with LC exhibited a protective effect similar to that of C75 (40 μM). We then examined mitochondrial function in LC-treated acinar cells. The reduction in OCR caused by STC was prevented by LC treatment; however, this protective effect was abolished upon the addition of Etomoxir (Supplementary Figure S3). As shown in Figures 4C–G, LC pretreatment significantly ameliorated the decrease in ΔΨm and ATP production, as well as the increase in mtROS and ox-mtDNA in STC-treated primary acinar cells. In contrast, inhibition of CPT1A by etomoxir effectively counteracted the protective effect of LC against the aforementioned mitochondrial dysfunction. LC promoted CPT1A expression, while etomoxir pretreatment significantly counteracted the inhibitory effect of LC on NLRP3 inflammasome activation, as evidenced by the levels of NLRP3, cleaved Caspase-1, GSDMD-NT, and IL-1β (Figures 4H–J; Supplementary Figure S7).

Taken together, these findings suggest that LC pretreatment exerts an effect similar to that of C75 in suppressing pancreatic acinar cell pyroptosis by activating CPT1A.

### 3.5 CPT1A activation ameliorates STC-induced pancreatic acinar cells injury

**FIGURE 5:**
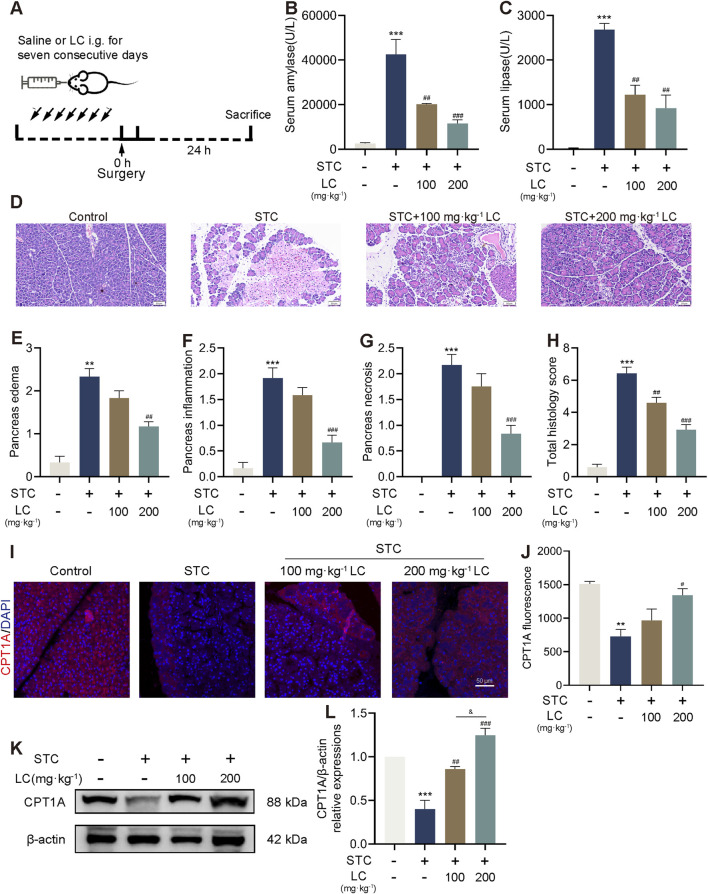
CPT1A activation ameliorates STC-induced pancreatic acinar cells injury. **(A)** The scheme for LC pretreatment of mice with SAP. **(B,C)** Serum amylase and lipase levels. **(D)** Representative H&E images. **(E–H)** Histological scores of the pancreas (edema, inflammatory infiltration, necrosis and total scores). **(I,J)** Immunofluorescence staining of CPT1A in the pancreas of mice received different treatments. Scale bar: 100 μm. **(K,L)** Protein levels of CPT1A and β-actin in the pancreas of mice received different treatments. All data are presented as means ± SEM, n = 3–5. **p < 0.01, ***p < 0.001 vs. Control group. ^#^ *p* < 0.05, ^##^ *p* < 0.01, ^###^ *p* < 0.001 vs. STC group.

Male C57BL/6 mice were treated with normal saline or varying doses of LC (100 and 200 mg/kg) for 7 consecutive days via gavage, followed by retrograde injection of 3% STC into the pancreatic duct to induce SAP (Figure 5A). The protective effect of LC pretreatment on STC-induced pancreatic injury was assessed by measuring serum levels of amylase and lipase, as well as evaluating pancreatic histological changes. Both low-dose (100 mg/kg) and high-dose (200 mg/kg) LC treatments significantly suppressed the increase in serum levels of amylase and lipase induced by STC injection (Figures 5B,C). Furthermore, histological improvements were observed in the high-dose LC-treated group, including reduced edema, inflammatory cell infiltration, and necrosis in the STC-injected pancreas (Figures 5D–H). Immunofluorescence analysis revealed a significant reduction in pancreatic CPT1A protein levels following STC injection, but this reduction was effectively reversed with LC pretreatment (Figures 5I,J). Western blotting confirmed a similar expression pattern for CPT1A (Figures 5K,L). In conclusion, these results suggest that LC pretreatment increases CPT1A expression in mice, providing a significant protective effect against acute biliary pancreatitis.

### 3.6 CPT1A protects against SAP in mice by inhibiting the NLRP3/GSDMD-mediated pyroptosis signalling pathway

**FIGURE 6:**
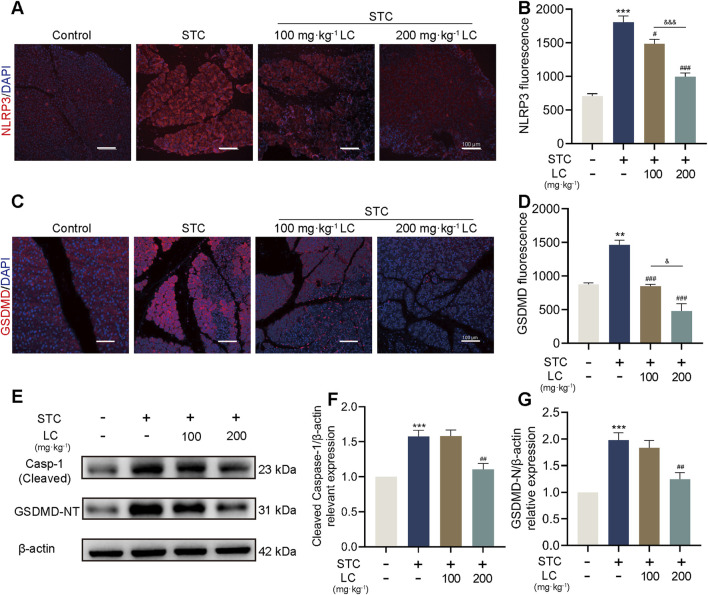
CPT1A protects against SAP in mice by inhibiting the NLRP3/GSDMD-mediated pyroptosis signalling pathway. **(A–D)** Immunofluorescence staining of NLRP3 and GSDMD in the pancreas of mice received different treatments. Scale bar: 100 μm. **(E–G)** Protein levels of cleaved Caspase-1 and GSDMD-NT in the pancreas of mice received different treatments. β-actin served as the loading control. All data are presented as means ± SEM, n = 3–5. *p < 0.05, **p < 0.01, ***p < 0.001 vs. Control group. ^#^ *p* < 0.05, ^##^ *p* < 0.01, ^###^ *p* < 0.001 vs. STC group. ^&^ *p* < 0.05, ^&&&^ *p* < 0.001 vs. LC (100 mg/kg) treatment group.

To investigate the protective mechanism of CPT1A in SAP, we assessed the expression changes of proteins involved in NLRP3 inflammasome activation. Immunofluorescence analysis revealed a marked increase in NLRP3 and GSDMD expression in the pancreas of STC-injected mice, which was significantly reduced by LC pretreatment (Figures 6A–D). Additionally, Western blotting data showed that the active forms of caspase-1 (cleaved casp-1) and GSDMD (GSDMD-NT) in pancreatic tissue were restored in STC-injected mice pretreated with high-dose LC (Figures 6E–G). Furthermore, treatment with 200 mg/kg of LC significantly suppressed IL-1β levels in the serum of mice (Supplementary Figure S10). Collectively, our results indicate that the protective mechanism of CPT1A against SAP primarily involves the inhibition of NLRP3 inflammasome activation and subsequent GSDMD-mediated pyroptosis.

## 4 Discussion

Over the past decade, a large number of studies have highlighted the protective role of CPT1A in inflammatory diseases. While the role of CPT1A in promoting FAO is well-known, the exact molecular mechanisms underlying its protective effects in pancreatitis remain largely unexplored. In the present study, we demonstrated that activation of CPT1A effectively reduced STC-induced acinar cell pyroptosis both *in vivo* and *in vitro*. This protective effect is attributed to the ability of CPT1A expression to improve mitochondrial function and inhibit the activation of the NLRP3 inflammasome. Moreover, we found that silencing Cpt1a using siRNA triggered the activation of the GSDMD-NT fragment.

Pyroptosis is a recently identified form of programmed cell death characterized by cell swelling, rupture, and the release of pro-inflammatory contents (Al Mamun et al., 2021; Rao et al., 2022). This type of cell death is implicated in various inflammatory diseases, such as sepsis (Zheng et al., 2021; Rao et al., 2022), cerebral hemorrhage (Yan et al., 2021; Song et al., 2022), and AP (Al Mamun et al., 2022). Recent studies have highlighted the crucial role of NLRP3 inflammasome in activating pancreatic acinar cell pyroptosis during AP (Fan et al., 2021; Ma et al., 2022). However, the precise mechanism triggering NLRP3 activation in acinar cells remains elusive. Mitochondrial dysfunction, particularly the overproduction of mtROS and the release of ox-mtDNA, is a major driver of NLRP3 inflammasome activation (Lawrence et al., 2022; Xian et al., 2022). Our study revealed that STC treatment decreased CPT1A expression in pancreatic acinar cells, which correlated with impaired mitochondrial function, including reduced ΔΨm and ATP production and increased mtROS and ox-mtDNA. Notably, C75 or LC pretreatment, which activate CPT1A, effectively preserved mitochondrial function and consequently reduced acinar cell pyroptosis. Thus, our findings suggest that the suppression of CPT1A expression following pancreatic injury may be the initial trigger for mitochondrial dysfunction and subsequent NLRP3 inflammasome-mediated pyroptosis.

CPT1A is the most widely distributed isoform of the CPT1 family, and its aberrant expression can lead to metabolic disorders and the progression of various cancers (Schlaepfer and Joshi, 2020; Liang, 2023). CPT1A deficiency results in the inability to generate acylcarnitines that enter the mitochondria for FAO, leading to impaired energy production (Raud et al., 2018a; Liang, 2023). Patients with CPT1A deficiency often experience multiple morbidities, including hepatic encephalopathy, hypoglycemia, and hyperammonemia (Tan et al., 2011; Raud et al., 2018a). Upregulation of CPT1A expression enhances FAO, which in turn increases intracellular NADPH levels, contributing to the attenuation of mtROS accumulation (Su et al., 2023). Elevated mtROS are known to trigger the release of oxidized ox-mtDNA, a key activator of the NLRP3 inflammasome pathway (Martinon, 2012; Jia et al., 2020). A recent study demonstrated an overproduction of mtROS and subsequent activation of NLRP3 inflammasome-mediated pyroptosis in microglia in an experimental model of hepatic encephalopathy (Cheon et al., 2023). Combining these findings with our study, it is reasonable to speculate that hepatic encephalopathy caused by CPT1A deficiency may be attributed to the activation of microglial pyroptosis. Furthermore, increased CPT1A expression has been observed in various cancers, such as breast, prostate, glioblastoma, leukemia, and colon cancers (Liang, 2023). Previous studies have already indicated the anti-apoptotic and anoikis resistance effects of CPT1A in mediating cancer cell survival and metastasis (Tian et al., 2022). Therefore, by promoting CPT1A-mediated FAO may be possible to interrupt this pro-inflammatory cascade and reduce pyroptotic cell death in acinar cells. In our study, etomoxir inhibited CPT1A activity and siRNA-mediated silencing of Cpt1a gene expression both enhanced NLRP3-mediated cellular pyroptosis. Meanwhile, C75, an agonist of CPT1A, reduced ΔΨm depolarisation, mtROS accumulation, and cellular pyroptosis-associated protein expression. Our findings here suggest that CPT1A activation may also exert an anti-pyroptotic effect, providing new insights into patient screening strategies for pyroptotic-targeted anticancer therapies.

C75 is a synthetic fatty-acid synthase (FASN) inhibitor and a potent CPT1A activator (Xue et al., 2021). Our study found that C75 can also promote the expression of CPT1A, possibly by inhibiting FASN and altering lipid metabolism, which activates transcription factors such as PPARα, thereby regulating the transcription of Cpt1a (Huang et al., 2013; Peng et al., 2024). However, further experiments are needed to validate our hypothesis regarding this mechanism. C75 is cytotoxic to many cell lines and this effect is thought to be mediated by the accumulation of malonyl coenzyme A in cells with an upregulated FASN pathway (Makowski et al., 2013). In our study, the concentration of C75 above 80 μM produced significant cytotoxicity to pancreatic acinar cells. Therefore, we would like to seek a stable CPT1A agonist which was safer. It has been shown that LC treatment increases both protein levels and activity of CPT1A, thereby improving mitochondrial function (Li et al., 2023). Our study further demonstrates that LC indeed promoted CPT1A expression, as reflected by a significant increase in CPT1A protein levels in LC-pretreated pancreatic acinar cells. The protective effect of LC in AP is largely attributed to the upregulation of CPT1A. This hypothesis was further supported by the fact that the inhibition of CPT1A by etomoxir counteracted the protective effect of LC pretreatment against oxidative damage to ΔΨm and mtDNA in STC-treated acinar cells. We also observed that LC inhibited acinar cell pyroptosis, including reduced expression of proteins related to the pyroptosis signalling pathway, which was reversed by etomoxir treatment. Thus, we tentatively put forward that CPT1A attenuates cell death by restoring mitochondrial function and by regulating NLRP3/GSDMD.

Although this study provides valuable insights into the role of CPT1A in the pathogenesis of SAP, several limitations should be acknowledged. Firstly, the STC-induced AP model used in our study was primarily designed to mimic cholestatic pancreatitis pathogenesis (Aho et al., 1980), there are other types of clinical AP, such as alcohol-origin pancreatitis, hypertriglyceridemia-AP, etc. Additionally, the STC-AP model differs from actual clinical cholestasis and cannot fully replicate human disease. We intend to further explore and validate the translational relevance of our findings in future studies. CPT1A expression levels are closely associated with the extent of FAO (Raud et al., 2018b), however, further investigations are needed to strengthen the credibility of our findings. In future studies, we plan to assess the changes in fatty acid profiles within our model, as well as evaluate the expression levels of other genes involved in FAO. This will reinforce the robustness of our conclusions.

In conclusion, our study found that promoting CPT1A expression ameliorated pancreatic acinar cell pyroptosis. At the molecular level, silencing or inhibiting CPT1A expression promoted the mtROS production, leading to the activation of NLRP3 inflammasome and GSDMD-NT, mitochondrial dysfunction, and ultimately aggravates pyroptosis of acinar cells. In contrast, pharmacological activation of CPT1A effectively mitigated pancreatic injury caused by STC-induced pyroptosis. These findings offer new insights into the complex mechanisms underlying acinar cell pyroptosis in SAP and highlight the therapeutic potential of targeting CPT1A in the treatment.

## Data Availability

The original contributions presented in the study are included in the article/Supplementary Material, further inquiries can be directed to the corresponding authors.
