# Supplementary material for: CPT1A mediated preservation of mitochondrial inhibits pyroptosis in pancreatic acinar cells
Source: Front Cell Dev Biol. 2025 Jul 11;13:1577669. doi: 10.3389/fcell.2025.1577669 (PMC12289652; doi:10.3389/fcell.2025.1577669)
Supplement: Supplementary file 1 [file DataSheet1.docx]

Supplementary Material

# Supplementary Figures and Tables

## Supplementary Figures

**
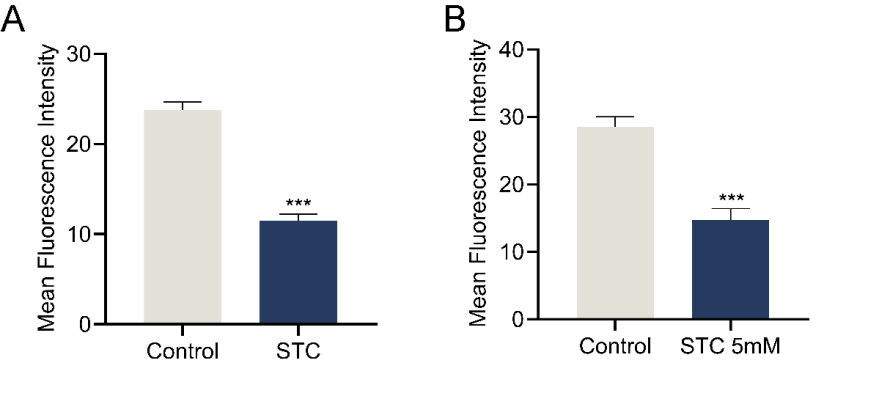
**

**Supplementary Figure 1. (A).** The quantification of CPT1A mean fluorescence intensity from three randomly selected fields per section (n = 3). **(B).** Quantification of CPT1A mean fluorescence intensity for the primary acinar cells from five randomly selected fields per group (n = 5). Data are presented as mean ± SEM. ^***^*p* < 0.001 vs. Control. Control: control group; STC: sodium taurocholate.

**
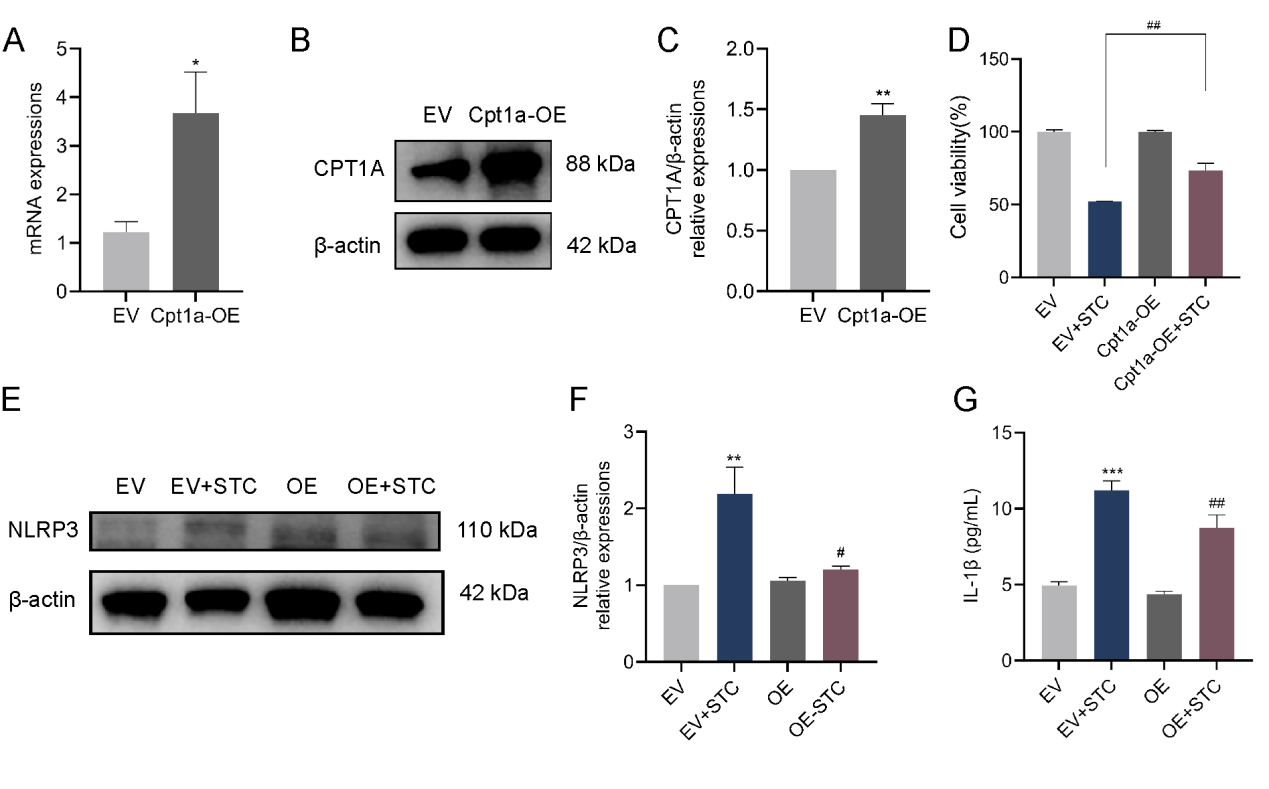
**

**Supplementary Figure 2. (A).** CPT1A mRNA relative expressions to EV (n = 4). **(B).** Representative image of CPT1A protein expression for EV group and Cpt1a-OE group. **(C).** The quantification of protein expression was normalized to β-actin levels. **(D).** CCK8 was used to determine the cell viability. **(E).** Representative image of NLRP3 protein expression for EV group, EV+STC group, Cpt1a-OE group and Cpt1a-OE+STC group. **(F).** Quantification of protein expression was normalized to β-actin levels. **(G).** 266-6 cells with either normal expression or Cpt1a-OE were treated with STC. After treatment, cell lysates were collected, and IL-1β levels were measured using ELISA. Data are presented as mean ± SEM from three independent experiments. ^*^*p* < 0.05, ^**^*p* < 0.01, ^***^*p* < 0.001 vs. EV group. ^#^*p* < 0.05, ^##^*p* < 0.01 vs. EV+STC group. EV: Empty vector group, Cpt1a-OE/OE: Cpt1a overexpression gruop, STC: sodium taurocholate.


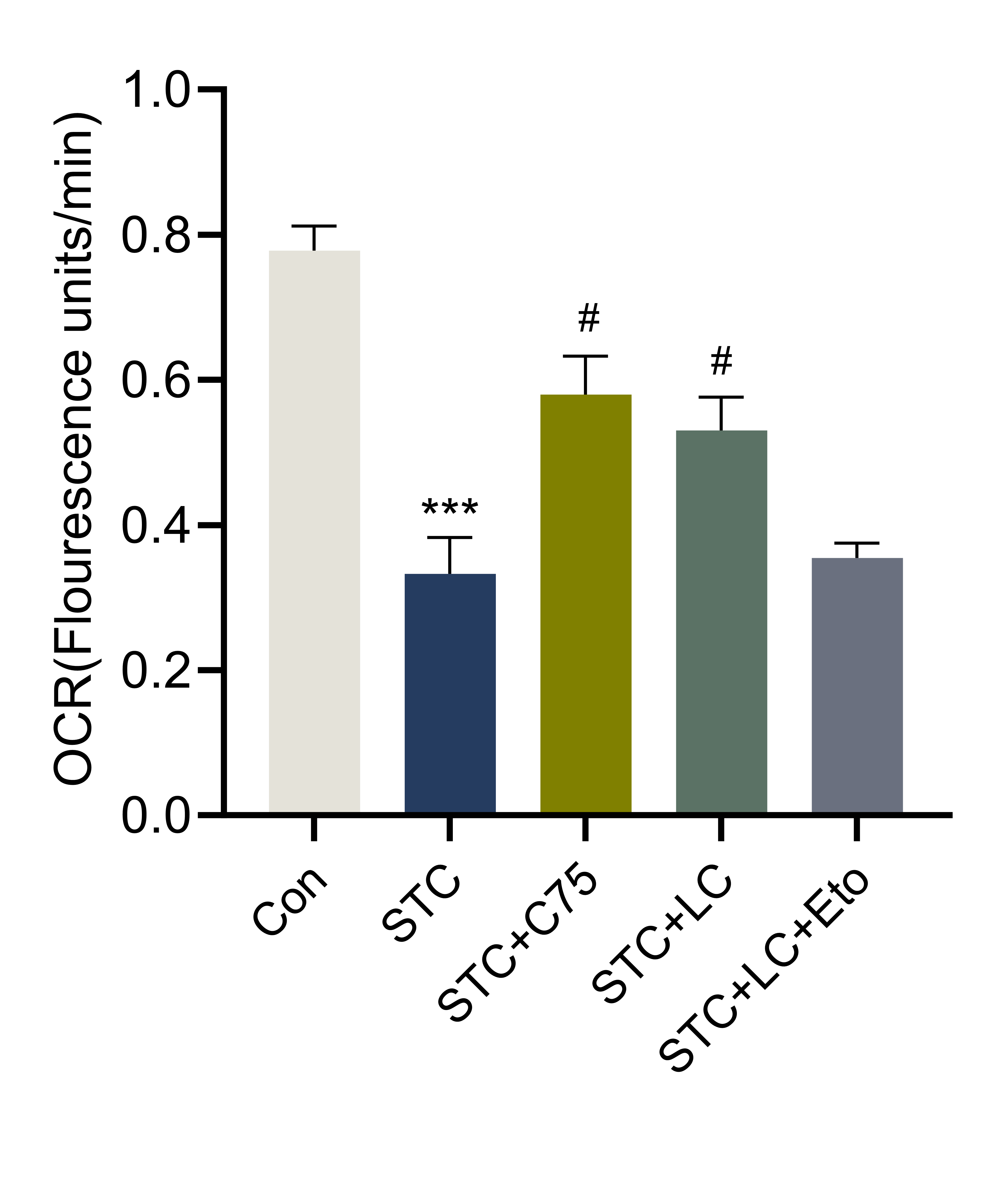


**Supplementary Figure 3.** After treatment, OCR were measured using assay kits from Elabscience according to the manufacturer's instructions. The bar graph displays OCR detection results, n = 4. Data are presented as mean ± SEM. ^***^*p* < 0.001 vs. Control group. ^#^*p* < 0.05 vs. STC group.


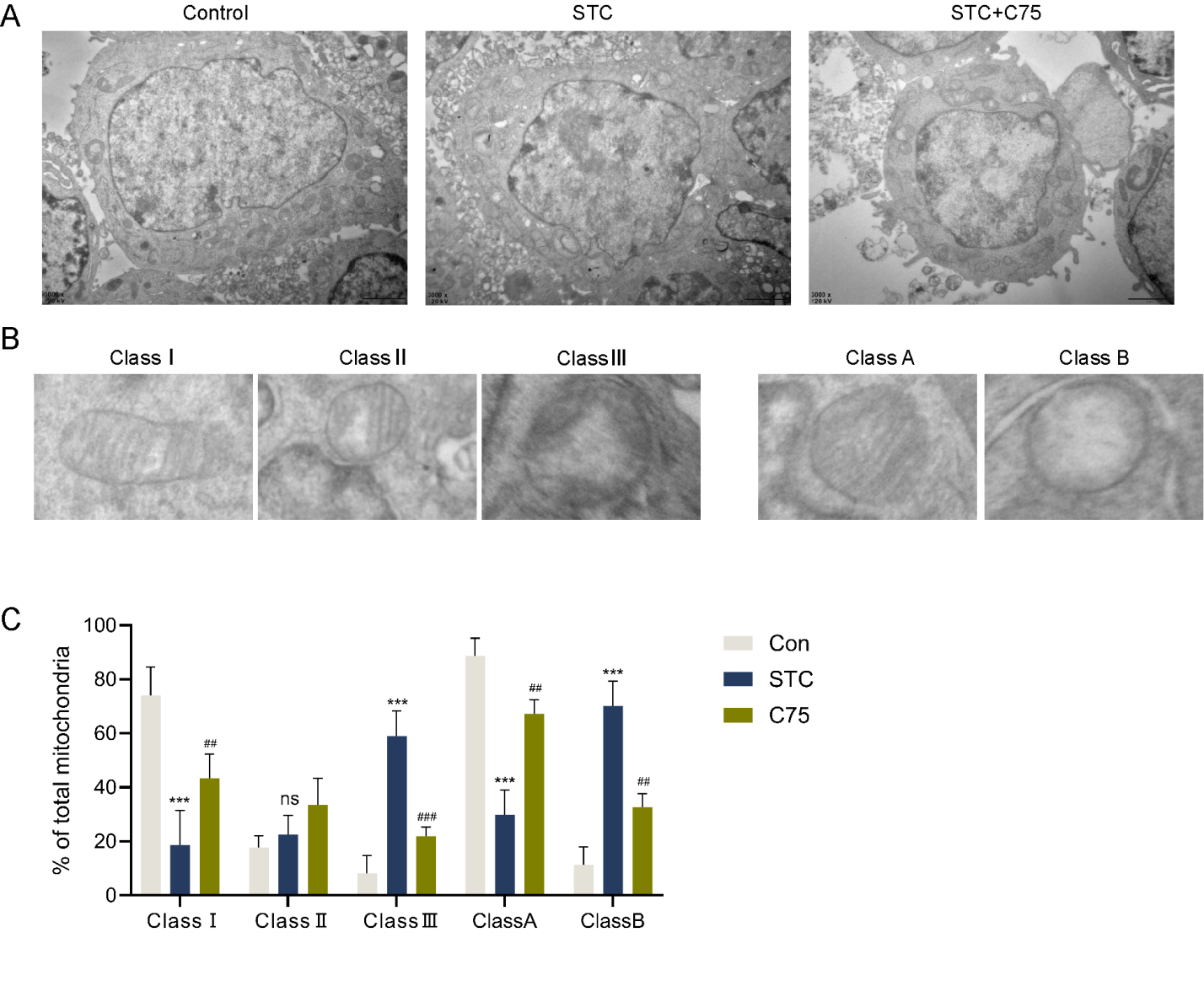


**Supplementary Figure 4. (A).** Representative transmission electron microscopy (TEM) images of 266-6 cells. **(B).** Mitochondria were grouped in accordance with mitochondrial crista number, matrix density, and swelling degree. **(C).** A total of 10–30 mitochondria per experiment were divided into three categories in accordance with crista number: Class I (more than four cristae), Class II (two or three cristae), and Class III (no more than one cristae); 10–30 mitochondria per experiment were divided into two categories in accordance with matrix density and swelling degree: Class A (mitochondria with a dense matrix) and Class B (mitochondria with a hypodense matrix). Scale bar: 2 μm. n = 5 in each group. Data are presented as mean ± SEM. ^***^*p* < 0.001 vs. Control group. ^##^*p* < 0.01, ^###^*p* < 0.001 vs. STC group.


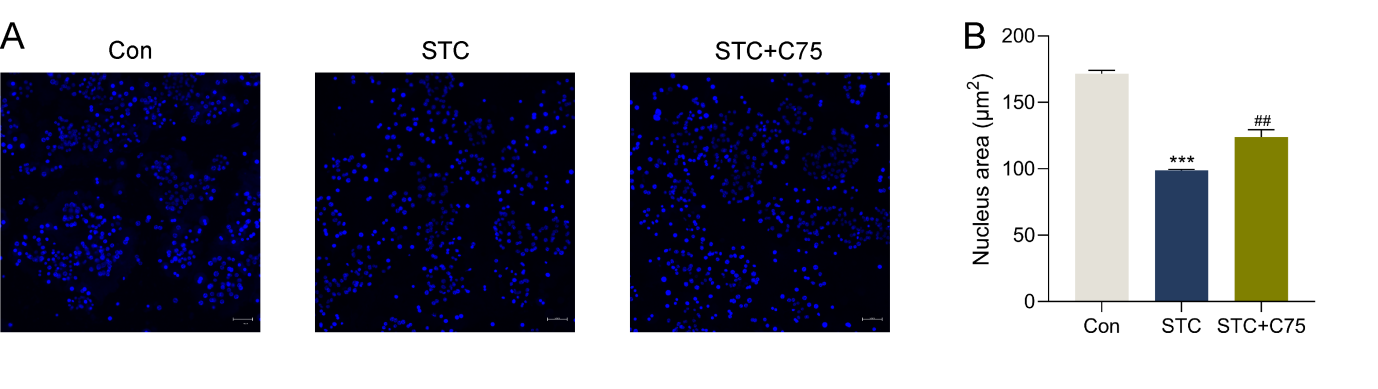


**Supplementary Figure 5.** (A) Representative images of Hoechst 33342 (blue) staining of primary acinar cells received different treatments. (B) At least three fields of view were selected for each group, and all nuclei in the selected fields were counted for nuclear area using ImageJ. All data are presented as means ± SEM. ^***^*p* < 0.001 vs. control group. ^##^*p* < 0.01 vs. STC group.


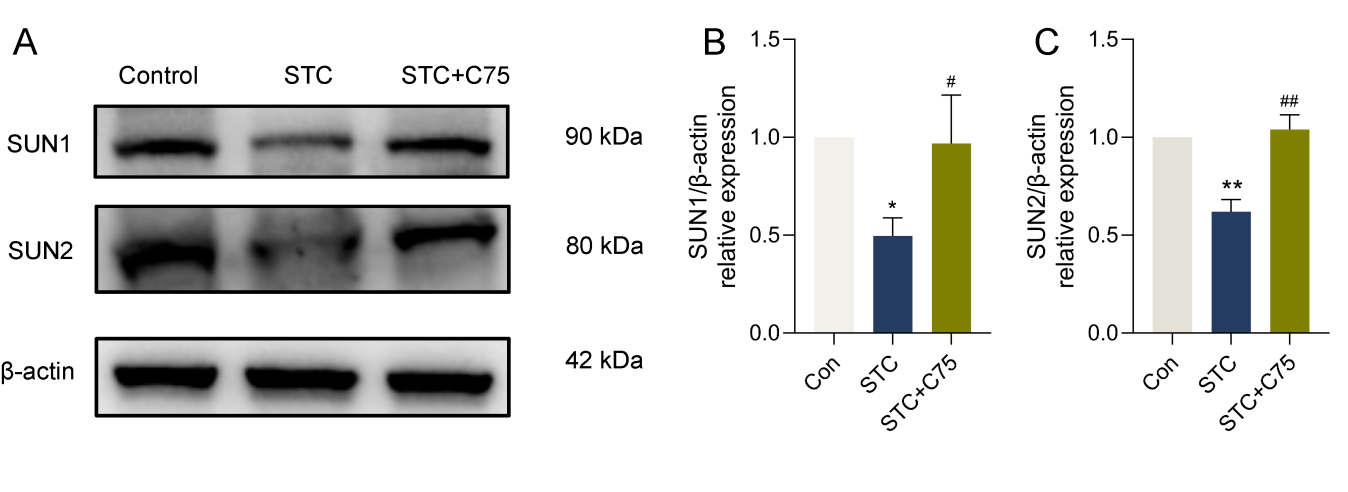


**Supplementary Figure 6.** Protein levels of SUN1, SUN2 and β-actin in the primary acinar cells received different treatments. All data are presented as means ± SEM. ^*^*p* < 0.05, ^**^*p* < 0.01 vs. control group. ^#^*p* < 0.05, ^##^*p* < 0.01 vs. STC group.


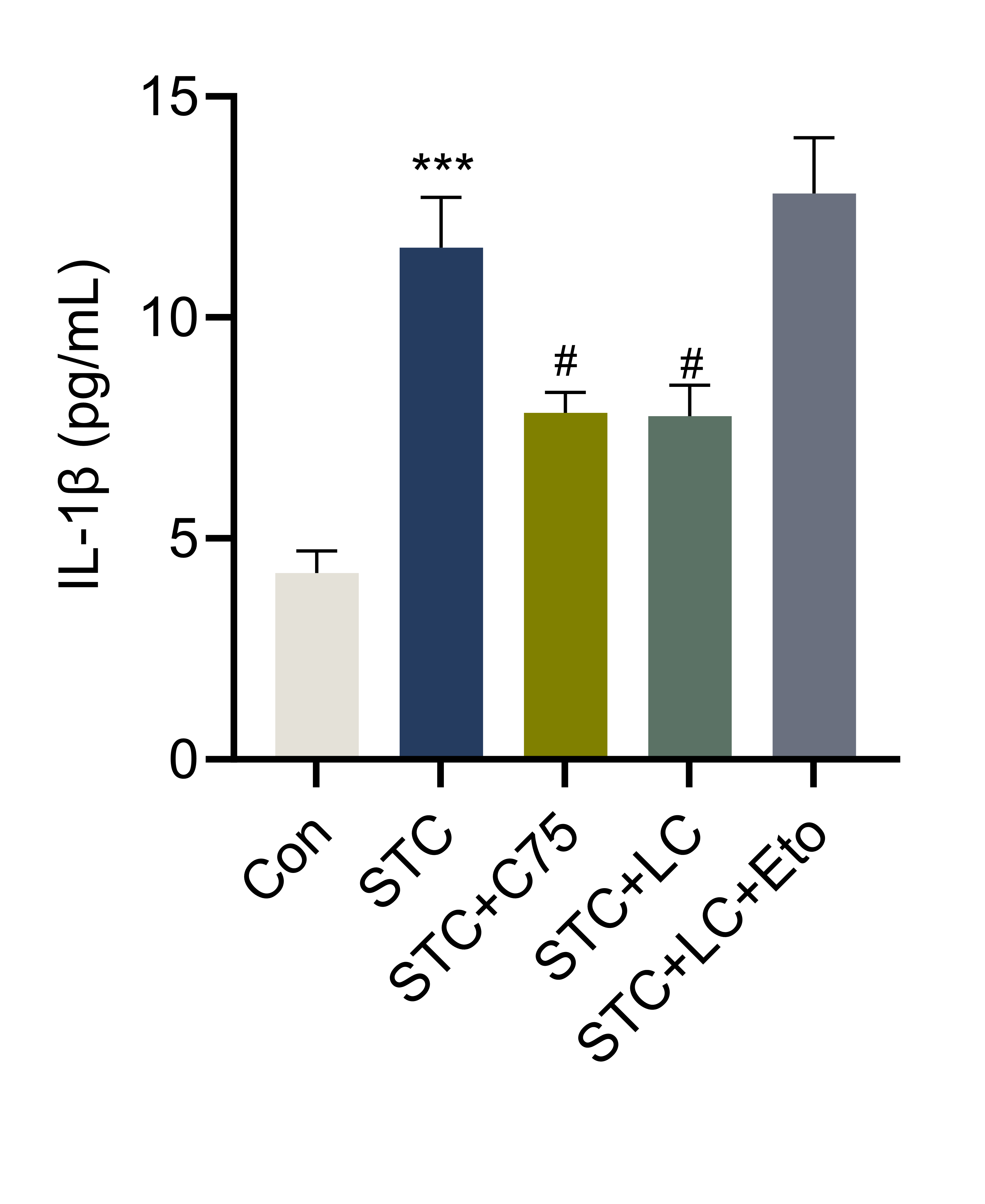


**Supplementary Figure 7.** After treatment, cell lysates were collected, and IL-1β levels were measured using ELISA. All data are presented as means ± SEM. ^***^*p* < 0.001 vs. control group. ^#^*p* < 0.05 vs. STC group.


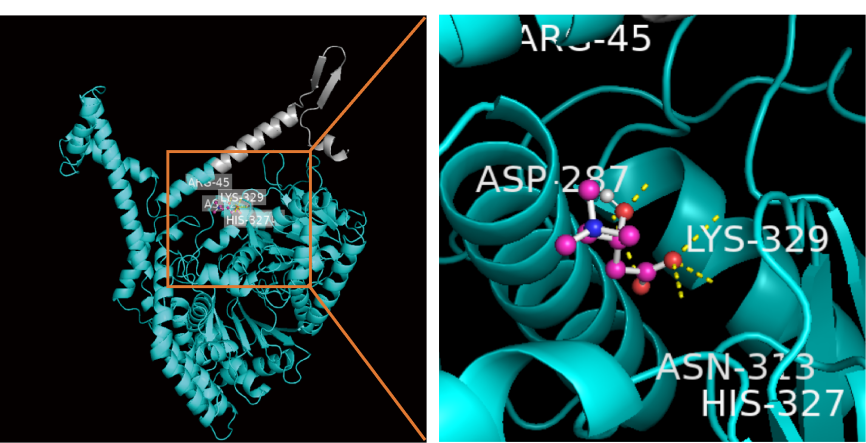


**Supplementary Figure 8.** The proposed binding model of LC to CPT1A based on docking studies. It shows that CPT1A interacts with LC through three potential active site amino acid residues, ARG45, ASP287, LYS329, ASN313 and HIS327.


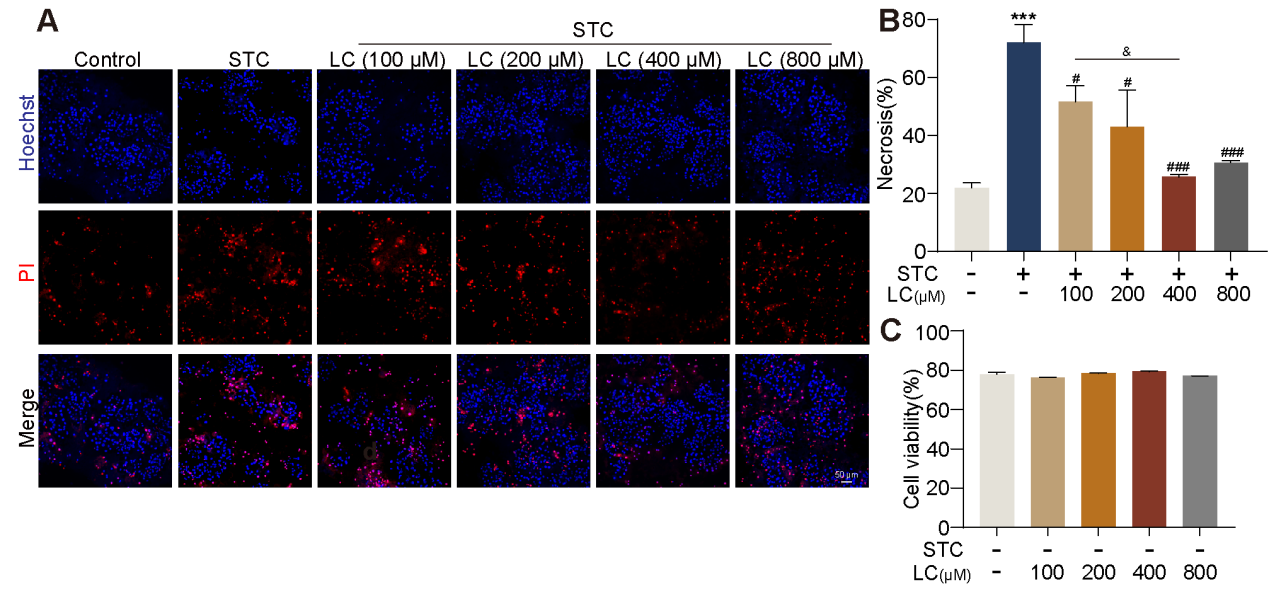


**Supplementary Figure 9.** LC treatment inhibits STC-induced primary acinar cells necrosis. (A) Representative image of Hoechst 33342 and PI staining of acinar cells (Scale bar: 50 μm). (B) The quantification of acinar cell necrosis. (C) LC showed no toxicity compared to the control group. All data are presented as means ± SEM, n = 3–5. ^***^p < 0.001 vs. control group. ^#^p < 0.05, ^###^p < 0.001 vs. STC group. ^&^p < 0.05 vs. LC (100 μmol·L^-1^) treatment group. STC: sodium taurocholate; LC: L-carnitine; PI: Propidium Iodide.


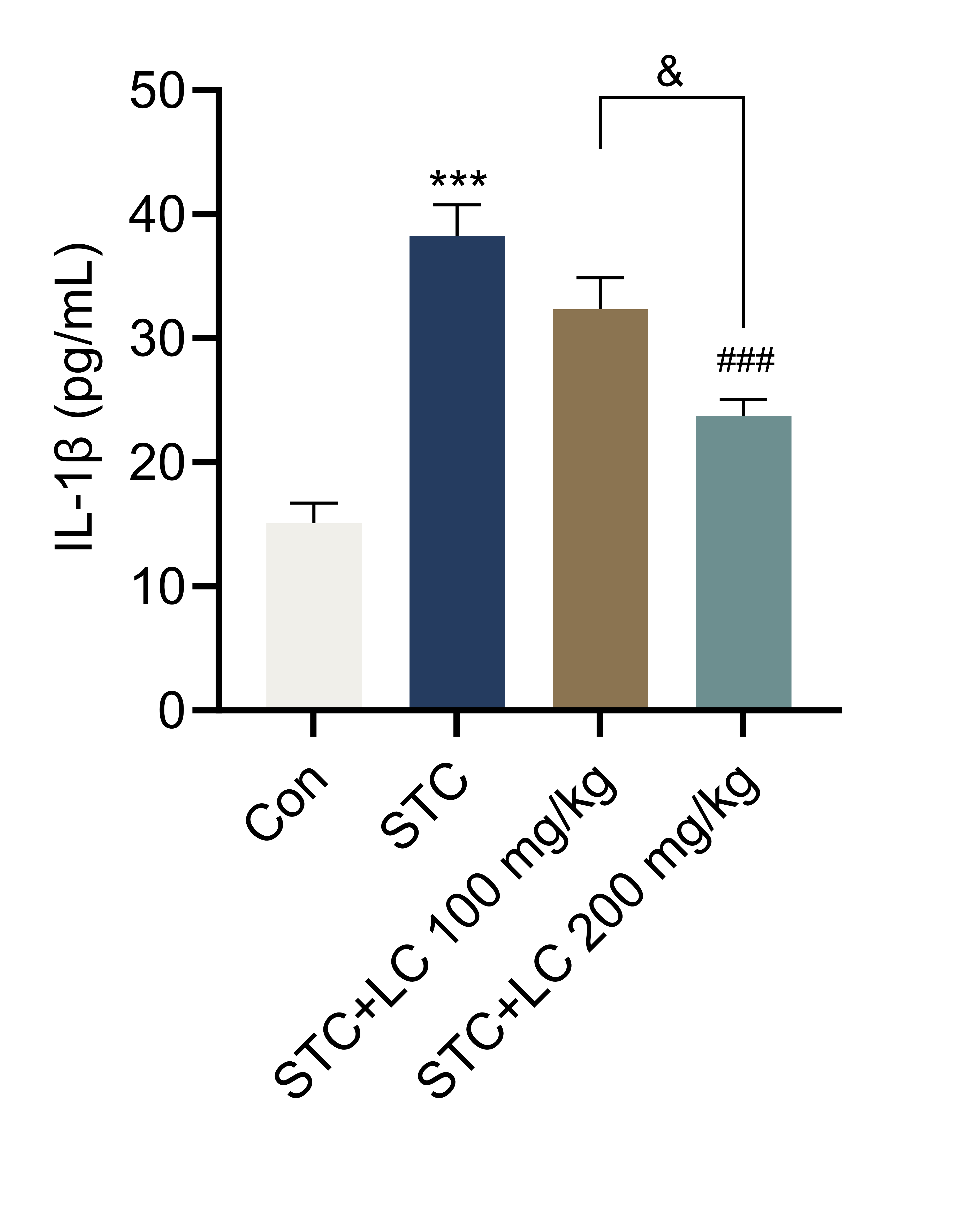


**Supplementary Figure 10.** The levels of serum IL-1β. All data are presented as means ± SEM, n = 3–5. ^***^p < 0.001 vs. control group. ^###^p < 0.001 vs. STC group. ^&^p < 0.05 vs. LC (100 mg/kg) treatment group. STC: sodium taurocholate; LC: L-carnitine.

## Supplementary Table

Supplementary Table 1. Score criteria of pancreatic injury.

| Parameter and score | Criterion |
| --- | --- |
| Edema 0 | No |
| Edema 1 | Widened area of interlobular space（< 20 %） |
| Edema 2 | Widened area of interlobular space（20 % − 50 %） |
| Edema 3 | Widened area of interlobular space（> 50 %）and the destruction and isolation of acinar cells |
| Inflammatory cell infiltration 0 | No |
| Inflammatory cell infiltration 1 | Inflammatory cells in or around the pancreatic ductal |
| Inflammatory cell infiltration 2 | Inflammatory cells in pancreas parenchyma（< 50 % pancreatic lobule） |
| Inflammatory cell infiltration 3 | Inflammatory cells in pancreas parenchyma（> 50 % pancreatic lobule） |
| Necrosis 0 | No |
| Necrosis 1 | Necrosis area around the pancreatic duct（< 50 %） |
| Necrosis 2 | Focal necrosis of pancreatic parenchyma（5 − 20 %） |
| Necrosis 3 | Extensive necrosis of pancreatic parenchyma（20 − 50 %） |
